# The roles of *Magnaporthe oryzae* avirulence effectors involved in blast resistance/susceptibility

**DOI:** 10.3389/fpls.2024.1478159

**Published:** 2024-10-09

**Authors:** Xin Liu, Xiaochun Hu, Zhouyi Tu, Zhenbiao Sun, Peng Qin, Yikang Liu, Xinwei Chen, Zhiqiang Li, Nan Jiang, Yuanzhu Yang

**Affiliations:** ^1^ Key Laboratory of Southern Rice Innovation & Improvement, Ministry of Agriculture and Rural Affairs, Yuan Longping High-Tech Agriculture Co., Ltd., Changsha, China; ^2^ State Key Laboratory of Hybrid Rice, Hunan Hybrid Rice Research Center, Changsha, Hunan, China; ^3^ Yuelushan Laboratory, Changsha, China; ^4^ College of Life Sciences, Hunan Normal University, Changsha, Hunan, China; ^5^ State Key Laboratory for Biology of Plant Diseases and Insect Pests, Institute of Plant Protection, Chinese Academy of Agricultural Sciences, Beijing, China

**Keywords:** rice, blast disease, *Magnaporthe oryzae*, AVR effector, R protein, resistance

## Abstract

Phytopathogens represent an ongoing threat to crop production and a significant impediment to global food security. During the infection process, these pathogens spatiotemporally deploy a large array of effectors to sabotage host defense machinery and/or manipulate cellular pathways, thereby facilitating colonization and infection. However, besides their pivotal roles in pathogenesis, certain effectors, known as avirulence (AVR) effectors, can be directly or indirectly perceived by plant resistance (R) proteins, leading to race-specific resistance. An in-depth understanding of the intricate AVR-R interactions is instrumental for genetic improvement of crops and safeguarding them from diseases. *Magnaporthe oryzae* (*M*. *oryzae*), the causative agent of rice blast disease, is an exceptionally virulent and devastating fungal pathogen that induces blast disease on over 50 monocot plant species, including economically important crops. Rice-*M. oryzae* pathosystem serves as a prime model for functional dissection of AVR effectors and their interactions with R proteins and other target proteins in rice due to its scientific advantages and economic importance. Significant progress has been made in elucidating the potential roles of AVR effectors in the interaction between rice and *M. oryzae* over the past two decades. This review comprehensively discusses recent advancements in the field of *M. oryzae* AVR effectors, with a specific focus on their multifaceted roles through interactions with corresponding R/target proteins in rice during infection. Furthermore, we deliberated on the emerging strategies for engineering R proteins by leveraging the structural insights gained from *M. oryzae* AVR effectors.

## Introduction

Plants, being sessile, are constantly besieged by a plethora of phytopathogens such as fungi, bacteria, viruses, oomycetes and nematodes, which are capable of causing extensive damage to agrosystems, ecosystems, and human livelihoods (Milgroom, 2015). Unlike animals, plants are devoid of an adaptive immune system and specialized mobile immune cells to fend off the numerous potential threats posed by these pathogens (Spoel and Dong, 2012). Instead, they have evolved a sophisticated two-tiered innate immune machinery, composed of pathogen/microbe-associated molecular patterns (PAMPs/MAMPs)-triggered immunity (PTI) and effector-triggered immunity (ETI), which is fundamental for their survival in nature (Jones and Dangl, 2006; Boller and Felix, 2009; Macho and Zipfel, 2014). Highly conserved PAMPs/MAMPs, such as bacterial flagellin, peptidoglycan (PGN), lipopolysaccharide (LPS) and fungal chitin, are recognized by plant cell surface-localized pattern-recognition receptors (PRRs). This recognition triggers basal immune responses known as PTI, which serves as the first tier of plant immunity thwarting pathogen proliferation (Nürnberger et al., 2004; Jones and Dangl, 2006; Block et al., 2008). Plant PRRs are classified as either transmembrane receptor-like kinases (RLKs) or receptor-like proteins (RLPs), which possess highly variable ectodomains for the detection of a wide range of ligands (Ngou et al., 2021). To circumvent PTI, adapted phytopathogens deliver an arsenal of virulence factors known as effectors into plant apoplast or cytoplasm, where they suppress immune responses and create a favorable niche for pathogenesis, leading to effector-triggered susceptibility (ETS) (Boller and He, 2009). As a counter response, plants employ intracellular immune receptors, called resistance (R) proteins, to detect certain pathogen effectors, referred to as avirulence (AVR) effectors, either through direct interactions or indirect interactions. This recognition triggers effector-triggered immunity (ETI), which represents the second tier of plant immunity (Dodds et al., 2018). Among the diverse types of R proteins, nucleotide-binding, leucine rich-repeat receptors (NLRs) represent the largest group and they share a multi‐domain architecture typically composed of a central nucleotide‐binding (NB‐ARC) domain, a C‐terminal leucine-rich repeat (LRR) domain, and either a coiled‐coil (CC) domain, RPW8-like CC domain, or a Toll/interleukin‐1 receptor (TIR) at N-terminus, and are thus called CNLs, RNLs or TNLs, respectively (Takken and Goverse, 2012). CNLs and RNLs are found in both dicot and monocot plant species, while TNLs are absent in monocots (Shao et al., 2016; Liu et al., 2021a). Notably, RNLs as helpers act downstream of sensor NLRs, transducing immune signals rather than sensing AVR effectors (Jubic et al., 2019). Consistent with their specific roles in immunity, RNLs are usually characterized by a relatively low copy number in plant genomes (Zhong and Cheng, 2016). Furthermore, many NLRs contain additional noncanonical domains called the integrated domains (ID), such as the heavy metal-associated (HMA) domain, BED domain, RIN4/NOI domain or WRKY domain, which serve as baits to trap AVR effectors or monitor their activities (Cesari et al., 2014; Wu et al., 2015a; Duxbury et al., 2016; Kroj et al., 2016; De la Concepcion et al., 2022; Shimizu et al., 2022). Direct binding of AVRs or AVR-host target complexes to these IDs results in NLR activation and initiation of immune responses (Cesari et al., 2014; Fujisaki et al., 2015, 2017; Cesari, 2018; De la Concepcion et al., 2022). NLRs can function as single entities, in pairs, or within intricate networks (Adachi et al., 2019). In comparison to PTI, ETI elicits stronger defense responses, and is usually associated with localized plant cell death, termed the hypersensitive response (HR), to limit the spread of phytopathogens into neighboring uninfected cells (Jones and Dangl, 2006; Duxbury et al., 2021). To counteract ETI, phytopathogens are subject to either loss of function or production of the altered forms of their AVR effectors under selective forces. These adaptations allow them to evade recognition by R proteins or the target proteins (Stergiopoulos and de Wit, 2009). The ‘zig-zag-zig’ model, which depicts the relationship between PTI, ETS, and ETI, is the most widely used and concise model to date (Jones and Dangl, 2006). However, this model is increasingly being challenged. Firstly, AVR effectors are not always detected by NLRs, but can also be recognized by PRRs (Thomas et al., 1997). Secondly, PTI and ETI were initially considered as two separate and sequential branches of the plant immune system mediated by different receptors with distinct ligands perceived and activation modes in the model, but they actually share many downstream immune responses, such as mitogen-activated protein kinase (MAPK) cascades activation, Ca^2+^ flux, reactive oxygen species (ROS) burst and phytoalexins production (Jones and Dangl, 2006; Tsuda and Katagiri, 2010; Yu et al., 2017; Lolle et al., 2020; Lu and Tsuda, 2021; Liu et al., 2023b). Recently, accumulating evidence has revealed crosstalk between ETI and PTI, indicating that these two branches of the immune system are not entirely independent. Instead, they can synergistically enhance each other, thereby eliciting more robust immune responses against pathogen infections (Ngou et al., 2021; Pruitt et al., 2021; Tian et al., 2021; Yuan et al., 2021). The findings imply a much more intricate and interconnected nature of plant immune responses than previously hypothesized.

Given the pivotal role of AVR effectors in adapted phytopathogens, a profound comprehension of their mode of action is potentially conductive to conceptualize novel strategies for sustainable management of plant diseases. In this review, taking the phytopathogenic fungus *Magnaporthe oryzae* (*M*. *oryzae*), the causal agent of globally important rice blast disease, as an example, we elaborate the dual nature of functions of AVR effectors in rice blast resistance/susceptibility. We present updated findings on the molecular interactions between *M*. *oryzae* AVR effectors and rice R/target proteins, as well as the underlying structural basis. We also present recent progress in genetic engineering of R proteins to produce robust resistance in rice based on the structural knowledge.

## *M*. *oryzae* and rice blast disease

Rice (*Oryza sativa* L.) is a staple cereal food crop for over 3.5 billion people around the world and sustainable rice production is crucial in ensuring global food security (Khush, 2013; Muthayya et al., 2014). Besides, rice cultivation is the major source of income and employment for more than 200 million smallholder farmers in rice-growing regions (Tonini and Cabrera, 2011). Over decades, rice production has witnessed a remarkable surge, attributed to the adoption of innovative agro-technologies including exploitation of semi-dwarf gene, utilization of heterosis and improvements in farming management practices (Ma and Yuan, 2015). In 2022, world rice production was approximately 776.5 million tons, marking a significant increase of 3.6 times compared to the production levels in 1961 (FAO, https://www.fao.org/faostat/en/). However, it is insufficient to meet the projected demands of continuously increasing global population, which is expected to reach 9.7 billion by 2050 (Hu et al., 2022a). This challenge is further exacerbated by the shrinkage of arable land and escalating influence of various biotic (pests, weeds, diseases, etc.) and abiotic factors (drought, cold, acidity, heat, salinity, etc.) (Sandhu et al., 2020). Among the biotic constraints, diseases caused by phytopathogens accounting for extensive yield losses represent a significant threat to rice production. A wide array of rice diseases caused by fungi, bacteria, viruses and nematodes have been recorded (Slough, 1985). Notably, blast disease, caused by the filamentous ascomycete fungus *Magnaporthe oryzae* B.C. Couch (anamorph: *Pyricularia oryzae* Cavara), is undoubtedly the most devastating disease of rice (Wang et al., 2014). It is also known as an ancient disease with records dating back to the 17th century in China (Couch et al., 2005). Nowadays, this notorious disease has a widespread distribution across rice-growing regions globally (Kato, 2001; Skamnioti and Gurr, 2009). Rice blast disease is responsible for average rice yield losses of about 10% to 30% per year, which could fulfill the annual rice consumption of 60 million people (Dean et al., 2005). Under favorable conditions, its regional epidemics can be more destructive, leading to yield loss up to 100% (Dean et al., 2012). In a survey from phytopathologists worldwide, *M*. *oryzae* was ranked first in the Top 10 scientifically and economically fungal pathogen list (Dean et al., 2012).

*M*. *oryzae* is a complex species with a broad host range. It is capable of plaguing more than 50 *Poaceae* and *Cyperaceae* species, including agriculturally important crop species such as rice (*Oryza sativa*), wheat (*Triticum aestivum*), maize (*Zea maydis*), barley (*Hordeum vulgare*), foxtail millet (*Setaria italica*) and finger millet (*Eleusine coracana*), as well as wild grasses such as weeping lovegrass (*Eragrostis curvula*), ryegrass (*Lolium perenne*) and goosegrass (*Eleusine indica*) (Khang et al., 2010; Hossain, 2022). Phylogenetic analyses have shown that *M*. *oryzae*’s wide host range is associated with intraspecific diversity (Couch et al., 2005; Chiapello et al., 2015; Yoshida et al., 2016; Inoue et al., 2017; Gladieux et al., 2018b). *M*. *oryzae* can be divided into several, genetically differentiated lineages that are associated with a specific or limited number of hosts (Gladieux et al., 2018a). All rice-infecting isolates (*Oryza* lineage) belong to a genetic lineage which is closely related to isolates infecting foxtail millet (*Setaria* lineage) (Couch et al., 2005; Ceresini et al., 2018; Gladieux et al., 2018a). Rice blast disease was thus inferred to emerge as a result of a host shift from foxtail millet in the Middle Yangtze Valley of China approximately 2,500 to 7,500 years ago (Couch et al., 2005). The globally distributed rice-infecting isolates can be further subdivided into four main lineages with one recombining lineage and three clonal lineages, which were estimated to have diverged around 1,000 years ago (Zhong et al., 2018; Gladieux et al., 2018a, b; Latorre et al., 2020; Thierry et al., 2022).

Almost all rice plant tissues at any growth stage can be attacked by this pathogen (Wilson and Talbot, 2009; Fisher et al., 2012). *M*. *oryzae* invades rice aerial tissues in a hemi-biotrophic manner, but it adopts a biotrophic strategy to infect roots (Marcel et al., 2010). During the infection of rice arial tissues, *M*. *oryzae* initially grows in living host cells as a biotroph to suppress the host immunity (Yan and Talbot, 2016). Subsequently, the invasive hyphae spread into neighboring cells through plasmodesmata and the fungus switches to a necrotrophic lifestyle. The initially infected host cells are destroyed, enabling the fungus to utilize nutrients released from the dead cells and sporulate from necrotic disease lesions on the leaf surface (Yan et al., 2023). The newly formed conidia are dispersed by wind or rain splashes, re-infecting healthy tissues and plants in the vicinity.

## Secretion of *M*. o*ryzae* effectors during host invasion

During the process of host invasion, *M*. *oryzae* undergoes several morphogenetic transitions. Initially, the three-celled conidium germinates to form a germ tube and differentiates into a dome-shaped infection structure called appressoria after perceiving physical and chemical cues on the leaf surface (Bourett and Howard, 1990). Subsequently, a penetration peg emerges from an appressorium for puncturing the host epidermal cell with huge turgor pressure and it then differentiates into the narrow tubular primary invasive hyphae (IH) and the bulbous secondary IH (Dagdas et al., 2012). The IH are enclosed by a host-derived plasma membrane termed the extra-invasive hyphal membrane (EIHM) (Kankanala et al., 2007). Once filled with the bulbous IH, the colonized host cells die. Meanwhile, the fungus protrudes into the neighboring host cells through pit field sites containing plasmodesmata, resulting in typical lesion formation and transition of the fungus from biotrophic to the necrotrophic phase (Martin-Urdiroz et al., 2016). During the biotrophic phase, *M*. *oryzae* express and secretes a set of effectors around or into host cells to modulate its cellular and metabolic processes, thereby favoring successful invasion and proliferation within plant tissues (Zhai et al., 2022). These effectors can thus be broadly categorized into apoplastic effectors and cytoplastic effectors based on their subcellular localizations in the host. Their deliveries are dependent on different secretion pathways (Mentlak et al., 2012; Yan and Talbot, 2016). Apoplastic effectors are delivered into the space between the fungal cell wall and host plasma membrane via a classical Golgi-dependent secretion pathway that can be blocked by the pharmacological drug brefeldin A (BFA) (Giraldo et al., 2013; Rocafort et al., 2020). Cytoplasmic effectors are secreted and accumulate in an extended dome-shaped interfacial region known as the biotrophic interfacial complex (BIC) near the tip of the first bulbous cell (Khang et al., 2010; Giraldo et al., 2013; Oliveira-Garcia et al., 2023). The cytoplasmic effectors within BIC are further packaged in dynamic vesicle-like membranous effector compartments (MECs), which are bounded by the host plasma membrane and CLATHRIN LIGHT CHAIN 1, a component of clathrin-mediated endocytosis (CME) (Oliveira-Garcia et al., 2023). Inhibition of CME by gene silencing or chemical treatments prevents MEC formation and pathogenicity, which indicates that CME facilitates the internalization of cytoplasmic effectors into host cells (Oliveira-Garcia et al., 2023). The emergence of the BIC structure is a feature of successful infection, but it is not observed during incompatible reactions (Mosquera et al., 2009; Khang et al., 2010; Jones et al., 2016; Shipman et al., 2017). Once internalized, these cytoplasmic effectors execute function in the cytoplasm and/or organelles of infected host cells, and even migrate to the adjacent cells (Khang et al., 2010).

## AVR effectors in *M*. *oryzae*

Molecular characterization of effectors stands as a fundamental step for understanding pathogen pathogenesis and plant immunity. Through genomic and transcriptomic analysis, researchers have pinpointed hundreds of potential effector candidates in *M*. *oryzae* (Dean et al., 2005; Soanes et al., 2008; Yoshida et al., 2009; Choi et al., 2010; Chen et al., 2013; Dong et al., 2015; Yan et al., 2023; Liu et al., 2023a). More than forty *AVR* genes have been genetically identified and 18 have been molecularly characterized thus far, including *PWL1*, *PWL2*, *PWT3*, *PWT4*, *PWT7*, *AVR-Rmg8*, *AVR-Pita*, *ACE1*, *AVR-Pia*, *AvrPii*, *AvrPiz‐t*, *Avr1‐CO39*, *AvrPib*, *AvrPi9*, *AvrPi54*, *AVR-Pias*, *AVR-Mgk1* and *Avr-Pik* (Kang et al., 1995; Sweigard et al., 1995; Orbach et al., 2000; Böhnert et al., 2004; Li et al., 2009; Yoshida et al., 2009; Ribot et al., 2013; Zhang et al., 2015; Wu et al., 2015b; Ray et al., 2016; Inoue et al., 2017; Anh et al., 2018; Zhang et al., 2020b; Shimizu et al., 2022; Asuke et al., 2023; Sugihara et al., 2023) (**Table 1**). *PWL2* encoding a glycine-rich, hydrophilic protein, is the first isolated *M*. *oryzae AVR* gene from *Oryzae* isolates (Sweigard et al., 1995). It belongs to a gene family with three other *PWL* (pathogenicity toward weeping lovegrass) genes (Kang et al., 1995). Both *PWL1* and *PWL2* are two host-specificity determinants conferring avirulence on weeping lovegrass but not on rice. However, *PWL3* and *PWL4* are nonfunctional. PWL2 is a core effector of the blast fungus, since it is ubiquitous in *M*. *oryzae* and has undergone substantial copy number expansion (Zdrzałek et al., 2024). PWT3, PWT4 and AVR-Rmg8 conditioning avirulence of *M*. *oryzae* isolates from different hosts on wheat, are able to trigger defense responses in wheat cultivars containing R proteins Rwt3, Rwt4 and Rmg7/Rmg8, respectively (Takabayashi et al., 2002; Vy et al., 2014; Inoue et al., 2017; Anh et al., 2018; Arora et al., 2023). *PWT3* homologs were found widely distributed across both *Triticum* and non-*Triticum* isolates, while *PWT4* homologs showed limited distribution in some isolates. Wheat cultivars without *Rwt3*, introduced to Brazil in the early 1980s, served as springboards for host jumps of *Lolium* isolates containing *PWT3* to wheat, followed by loss of function of *PWT3* due to the imposed selection by cultivars with *Rwt3* and wheat blast epidemics in South America, Asia as well as Africa (Inoue et al., 2017). *PWT7* from an *Avena* isolate confers avirulence on wheat only at the seedling stage (Asuke et al., 2023). *AVR-Rmg8*, identified from a *Triticum* isolate, was found to be recognized by either *Rmg7* in tetraploid wheat or *Rmg8* in hexaploid wheat, conferring resistance at both the seedling and heading stage (Anh et al., 2018). Among the 12 other *AVR* genes displaying avirulence toward rice, ten code for small proteins less than 200 amino acids (aa) with N-terminal signal peptides and share low sequence similarity to other proteins of known function in public databases. *ACE1* and *AVR-Pita* are the two exceptions, which encode larger proteins and contain known-function domains or motifs. *ACE1* is a secondary metabolism (SM) gene encoding a non-secreted PKS-NRPS hybrid (polyketide synthase and non-ribosomal peptide synthetase) enzyme (Böhnert et al., 2004). *AVR-Pita* encodes a putative neutral zinc metalloprotease (Orbach et al., 2000). The genetic instability of *AVR* genes in *M*. *oryzae* is considered to be a common mechanism in gaining virulence and causing rapid resistance erosion of their cognate *R* genes (Huang et al., 2014). Different mechanisms including insertion, point mutation, and deletion, as well as sexual mating and parasexual recombination are responsible for the loss of avirulence function of *AVR* genes (Noguchi et al., 2006; Tsujimoto Noguchi, 2011; Hu et al., 2022b). Among the cloned *AVR* genes of *M*. *oryzae*, *AVR*-*Pita* has been widely studied due to its relatively high variability. For example, *AVR*-*Pita* was found to be almost or completely absent in over half of the blast isolates in the Sichuan Basin, China, and five haplotypes with avirulent function were identified (Hu et al., 2022b). In an investigation of *M*. *oryzae* isolates from Thailand, *AVR*-*Pita* was detected in only around one third of them and six haplotypes of were identified with one deletion and 12 amino acid substitutions (Sutthiphai et al., 2022). Additionally, 40 *AVR*-*Pita* haplotypes were identified in avirulent isolates collected from Southern US (Zhang et al., 2020b). In contrast, *AVR*-*Pi9* is much more stable and it can be detected in all the *M*. *oryzae* samples in Sichuan and Yunan province, China, as well as Thailand (Hu et al., 2022b; Sutthiphai et al., 2022; Lu et al., 2023). Sequence analysis indicated that *AVR*-*Pi9* had a relatively low genetic diversity (Sutthiphai et al., 2022; Lu et al., 2023).

**Table 1:** AVR effectors identified in *M*. oryzae and their cognate R/target proteins in the hosts.

AVR effector	Protein Size (aa)	Encoding protein	Origin	Secretion site	Corresponding R protein	Host	Encoding protein	Interaction	Guardee/decoy/cofactors/interacted protein	Reference
PWL1	147	/	*Eleusine* isolate	BIC	/	Weeping lovegrass	/	/	/	Kang et al., 1995; Khang et al., 2010
PWL2	145	MAX	*Oryzae* isolate	BIC	/	Weeping lovegrass	/	/	OsHIPP43	Sweigard et al., 1995; Brabham et al., 2024; Were et al., 2024; Zdrzałek et al., 2024
					Mla3	Barley	CNL	/		
PWT3	141	/	*Avena* isolate	/	Rwt3	Wheat	CNL	/	/	Inoue et al., 2017; Arora et al., 2023
PWT4	93	/	*Avena* isolate	/	Rwt4	Wheat	WTK	/	/	
PWT7	98	/	*Avena* isolate	/	/	Wheat	/	/	/	Asuke et al., 2023
AVR-Rmg8	109	/	*Triticum* isolate	/	Rmg7	Wheat	RLK	/	/	Anh et al., 2018; Asuke et al., 2024
				/	Rmg8	Wheat	RLK	/	/	
AVR1-CO39	89	MAX	*Oryzae* isolate	EIHM	RGA4/RGA5	Rice	CNL/CNL-HMA	Direct	/	Okuyama et al., 2011; Cesari et al., 2013; Ribot et al., 2013
ACE1	4035	PKS-NRPS enzyme	*Oryzae* isolate	/	Pi33(t) (uncharacterized)	Rice	/	/	/	Böhnert et al., 2004; Collemare et al., 2008
AVR-Pii	70	ZiF	*Oryzae* isolate	BIC	Pii-1/Pii-2	Rice	CNL/CNL-RIN4/NOI	Indirect	OsExo70F3, OsNADP‐ME2	Yoshida et al., 2009; Fujisaki et al., 2015; Singh et al., 2016
AVR-Pita	223	Zinc metalloprotease	*Oryzae* isolate	BIC	Ptr	Rice	CNL	Indirect	OsCOX11	Zhao et al., 2018; Meng et al., 2020; Han et al., 2021; Xiao et al., 2024
AvrPiz-t	108	MAX	*Oryzae* isolate	BIC	Piz-t	Rice	CNL	Indirect	APIP4, APIP5, APIP6, APIP7, APIP10, APIP12	Li et al., 2009; Park et al., 2012, 2016; Wang et al., 2016; Tang et al., 2017; Shi et al., 2018; Zhang et al., 2020a
AVR-Pik alleles	113	MAX	*Oryzae* isolate	BIC	Pik-1/Pik-2 and alleles	Rice	CNL-HMA/CNL	Direct	OsHIPP20, AKIP30, WG7	Yoshida et al., 2009; Oikawa et al., 2024; Guo et al., 2024; Yang et al., 2024
AVR-Pia	85	MAX	*Oryzae* isolate	BIC	RGA4/RGA5	Rice	CNL/CNL-HMA	Direct	/	Yoshida et al., 2009; Okuyama et al., 2011; Cesari et al., 2013; Sornkom et al., 2017
AvrPi9	91	/	*Oryzae* isolate	BIC	Pi9	Rice	CNL	Indirect	ANIP1, OsWRKY62, OsRGLG5	Wu et al., 2015b; Shi et al., 2023; Liu et al., 2023c
AvrPib	75	MAX	*Oryzae* isolate	BIC	Pib	Rice	CNL	Indirect	SH3P2	Zhang et al., 2015, 2018; Xie et al., 2022
AvrPi54	153	/	*Oryzae* isolate	/	Pi54	Rice	CNL	Direct	/	Ray et al., 2016
AVR-Mgk1	85	MAX (predicted)	*Oryzae* isolate	/	Piks-1/Piks-2 and alleles	Rice	CNL-HMA/CNL	Direct	/	Sugihara et al., 2023
AVR-Pias	91	/	*Oryzae* isolate	/	Pias-1/Pias-2	Rice	CNL/CNL-DUF761	Indirect	/	Shimizu et al., 2022

/, not determined; BIC, biotrophic interfacial complex; EIHM, extra-invasive hyphal membrane; CNL, coiled-coil, nucleotide-binding, leucine rich-repeat receptors; WTK, wheat tandem kinase; RLK, receptor-like kinase; HMA, heavy metal-associated domain; RIN4/NOI, nitrate-induced/RPM1-interacting protein 4 domain; DUF761, domain of unknown function 761.

## Molecular interactions between *M. oryzae* AVR effectors and rice R/target proteins

**Figure 1:**
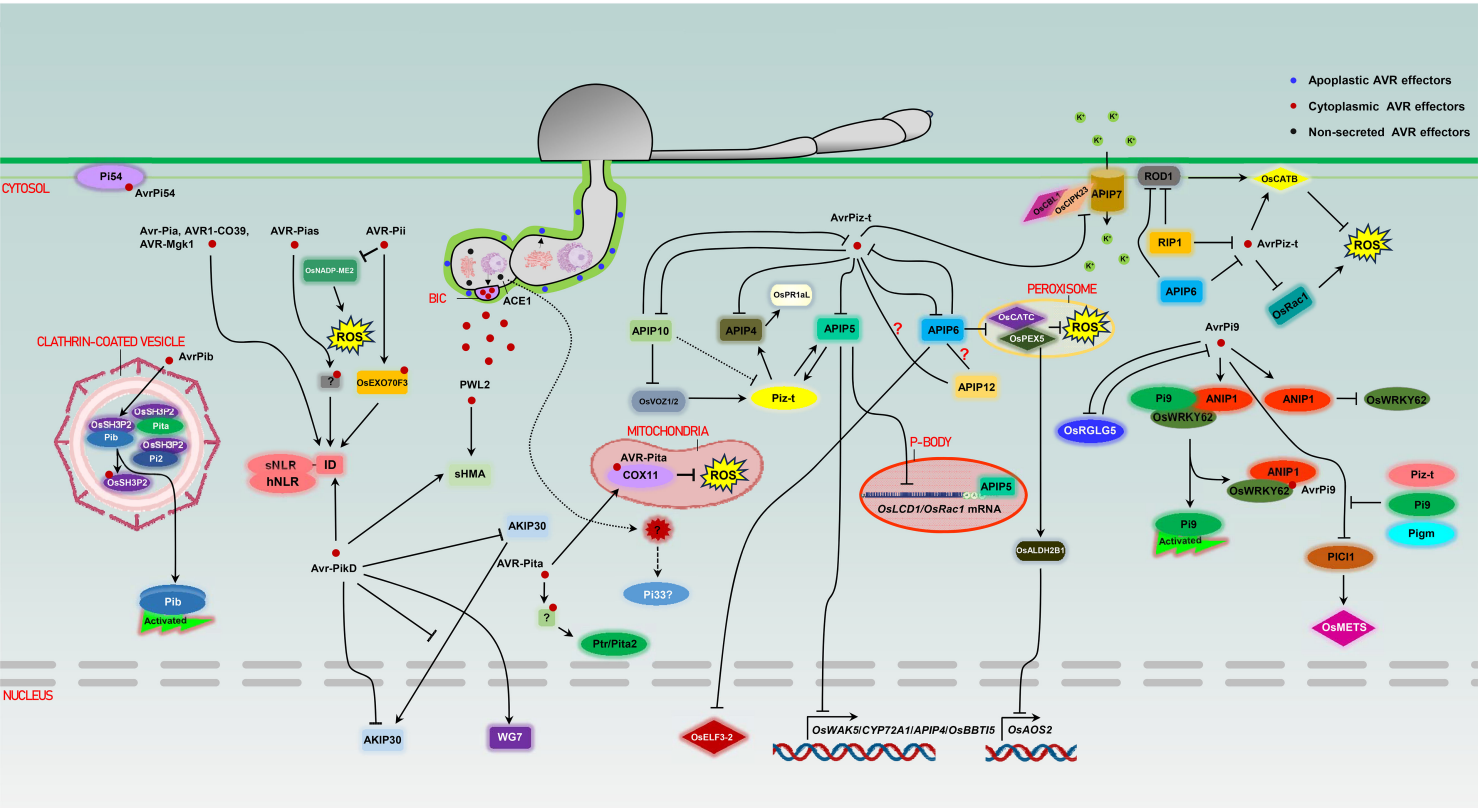
Molecular interactions between *M*. *oryzae* AVR effectors and R/target proteins in rice.

The detection of AVR effectors by cognate R proteins occurs via either direct or indirect interactions in Rice-*M*. *oryae* and other pathosystems (Stergiopoulos and de Wit, 2009). Direct recognition depends on physical binding of AVR effectors to the R proteins and indirect recognition involves the perception of effector-induced modifications of other host targets (usually termed guardees or decoys) by R proteins. It is considered that guardees play certain roles in plant immunity, while decoys specialize in trapping effectors without immune function (Van der Hoorn and Kamoun, 2008; Khan et al., 2016; Ao and Li, 2022). NLRs, as the most prevalent group of characterized R proteins in rice, function as singletons or pairs (Xi et al., 2022). Among over 40 cloned rice *R* genes, only *Pid-2*, *pi21*, *Ptr*, *Pi65* and *Pb4* encode non-NLR proteins (Devanna et al., 2022; Shimizu et al., 2022; Xiao et al., 2023; Fan et al., 2024). Most of the rice NLR pairs are genetically linked in a head-to-head orientation. One containing a noncanonical ID acts as a sensor NLR (sNLR) to directly detect the presence of the AVR effector(s), whereas the other one is a canonical NLR acting as a helper (hNLR) to transduce signals to activate immunity (Lüdke et al., 2022; Cadiou et al., 2023; Contreras et al., 2023). Singleton NLRs are capable of mediating both AVR effectors perception and downstream defense signaling initiation without relying on partner NLRs. Recent studies have revealed an extremely complex picture of *M*. *oryzae* AVR effectors and rice R/target proteins (**Figure 1**).

## AVR effector vs. singleton NLR

Pita-AVR-Pita is the earliest studied pair of R-AVR in rice-*M*. *oryzae* pathosystem and has long been accepted as a classic example of direct AVR effector binding by NLR (Bryan et al., 2000; Jia et al., 2000; Orbach et al., 2000). The mature form of AVR-Pita containing 176 aa at the C-terminus was found to bind specifically to the LRR region of Pita (Orbach et al., 2000). Single amino acid substitutions in either the LRR region of Pita or protease motif of AVR-Pita disrupt the physical interaction, resulting in failure of defense response initiation. *Ptr/Pita2* was later found to be not only closely linked to *Pita* but also involved in *Pita*-mediated resistance (Zhao et al., 2018; Meng et al., 2020). *Ptr*/*Pita2* encodes an atypical R protein with four Armadillo (ARM) repeats and its disruption leads to a loss of resistance to some AVR-Pita containing isolates, suggesting that it is required for the complete function of Pita. However, a very recent study indicated that Pita is involved in neither Pita resistance nor AVR-Pita detection (Xiao et al., 2024). It also has no role in Ptr/Pita2-mediated resistance. The Pita resistance is indeed provided by one of Ptr alleles, designated PtrB, which recognizes a restricted set of AVR-Pita alleles through an indirect way. PtrA can detect all natural AVR-Pita alleles and confers Pita2 resistance. Additionally, AVR-Pita was found to target OsCOX11, a cytochrome C oxidase (COX) assembly protein, in rice mitochondria (Han et al., 2021). OsCOX11 participates in ROS metabolism and plays a negative role in rice resistance. The AVR-Pita-OsCOX11 interaction increases the COX activity in ROS metabolism, thereby inhibiting ROS accumulation and suppressing rice innate immunity. Pi54-AvrPi54 is currently the only case of direct interaction between AVR effector and singleton NLR (Ray et al., 2016). Pi54 physically interacts with AvrPi54 at the host plasma membrane, which restricts the movement of AvrPi54 into nucleus for its virulence function (Saklani et al., 2023).

Three other singleton NLRs Pib, Pi9 and Piz-t recognize their cognate AVR effectors AvrPib, AvrPi9 and AvrPiz-t, respectively, via indirect way. SH3P2, an SH3 domain-containing protein mediates indirect AvrPib-Pib recognition (Xie et al., 2022). SH3P2 functions as a ‘‘protector’’ to associate with Pib mainly at clathrin-coated vesicles (CCV) in rice cells, which is an important coated vesicle responsible for endocytosis and many post-Golgi trafficking processes. The SH3P2-Pib association interferes with the Pib homodimerization by disrupting CC domain self-association, thus maintaining Pib in static state under normal growth conditions. Since SH3P2 associates with CCVs, which suggests SH3P2 may possess conserved intracellular trafficking functions and transfer Pib cargo to vacuoles for degradation, thereby maintaining a low abundance of Pib in the absence of blast fungus infection. Interestingly, it was found that SH3P2 also associates with the CC domains of Pi2 and Pita, but it is unclear whether SH3P2 affects resistance mediated by these two R proteins. SH3P2 can also bind to AvrPib in CCVs with higher affinity. Upon invasion of *M*. *oryzae* isolates containing AvrPib, the competitive binding of AvrPib to SH3P2 releases Pib from the OsSH3P2-Pib complex and alleviates the inhibition of Pib homodimerization, thus eventually activates Pib-mediated resistance. ANIP1-OsWRKY62 module was recently found to be targeted by AvrPi9 and regulates rice immunity in the presence/absence of Pi9 in distinct ways (Shi et al., 2023). ANIP1 is a rice ubiquitin-like domain-containing protein (UDP) subjected to 26S proteasome-mediated degradation. Both AvrPi9 and Pi9 can directly interact with and stabilize ANIP1 through disturbing its degradation. Moreover, ANIP1 physically interacts with the rice WRKY transcription factor OsWRKY62 and affects its abundance by promoting its degradation. OsWRKY62 was also found to interact with AvrPi9 and Pi9. In the absence of Pi9, lower abundance of ANIP1 leads to OsWRKY62 accumulation in rice plants and enhanced immunity during infection by *M*. *oryzae* isolates without AvrPi9. When infected by *M. oryzae* isolates with AvrPi9, ANIP1 is stabilized by AvrPi9 that more efficiently promotes the degradation of OsWRKY62, thus decreasing the immune response. In the presence of Pi9, it binds to and stabilize ANIP1-OsWRKY62 module. They form a complex with unknown adaptor(s) to maintain Pi9 in its inactive state under normal growth conditions. Under invasion by non-AvrPi9 *M*. *oryzae* isolates, the forming complex decreases plant immunity. Under invasion by *M*. *oryzae* isolates carrying AvrPi9, AvrPi9 promotes the dissociation of ANIP1 from Pi9, which further activate Pi9 and downstream immune responses. In contrast to ANIP as a negative regulator of rice immunity, the AvrPi9 interacting proteins OsRGLG5 and PICI1 were found to positively regulate rice defense (Zhai et al., 2022; Liu et al., 2023c). Both of these two proteins can be targeted for degradation. OsRGLG5, encoding a functional RING-type E3 ubiquitin ligase, functions as a positive regulator of basal resistance, but it is not required for Pi9-mediated blast resistance and no physical interaction between OsRGLG5 and Pi9 was observed, which suggested that OsRGLG5 may not be a guardee in the Pi9-AvrPi9 interaction (Liu et al., 2023c). In response, OsRGLG5 ubiquitinates and subsequently degrades AvrPi9 through the 26S proteasome pathway. The deubiquitinase PICI1, acts as an immune hub for both PTI and ETI through the methionine-ethylene cascade (Zhai et al., 2022). AvrPi9 was found to promote PICI1 degradation in a proteasome-independent manner, which in turns promotes methionine synthases OsMETS1 and OsMETS2 ubiquitination and degradation, leading to reduced methionine and ethylene biosynthesis, as well as comprised PTI. While NLRs, such as PigmR, Pi9 and Pizt, protect PICI1 from AvrPi9 binding in a competitive manner to reboot the methionine-ethylene-mediated immunity. AvrPiz-t was reported to target 12 APIPs (AvrPiz-t interacting proteins) in rice and the immune functions of several APIPs including APIP4 (Bowman-Birk trypsin inhibitor protein), APIP5 (bZIP transcription factor), APIP6 (Ring type E3 ubiquitin ligase), APIP7 (plasma membrane potassium channel), APIP10 (RING-type E3 ubiquitin ligases) and APIP12 (homologue of nucleoporin protein Nup98) have been well characterized (Park et al., 2012, 2016; Wang et al., 2016; Tang et al., 2017; Shi et al., 2018; Zhang et al., 2020a). AvrPiz-t can block the E3 ligase activity of APIP6 and APIP10 to suppress rice PTI (Park et al., 2012, 2016). In return, these two E3 ligase ubiquitinate and degrade AvrPiz-t to reduce its suppressive effects on rice PTI. APIP10 also promotes degradation of the Piz-t protein through the 26S proteasome system, although no direct interaction between these two proteins were observed. During *M*. *oryzae* infection, AvrPiz-t interferes the negative regulation of APIP10 on Piz-t, which leads to rapid accumulation of Piz-t protein and initiation of ETI (Park et al., 2016). The transcription factors OsVOZ1 and OsVOZ2 were found to bridge the connection between APIP10 and Piz-t (Wang et al., 2021). They function synergistically to negatively regulate basal defense but positively regulate Piz-t-mediated immunity. ROD1 is a C2 domain Ca^2+^ sensor, which recruits catalase CatB (OsCATB) to increase its activity for ROS elimination. AvrPiz-t structurally mimics ROD1 and executes similar ROS-scavenging-mediated immune suppression (Gao et al., 2021). But both ROD1 and AvrPiz-t can be targeted for ubiquitin-mediated degradation by APIP6 and the other E3 ligase RIP1. Besides, APIP6 can also ubiquitinate the catalase OsCATC, the peroxisomal receptor protein OsPEX5, and OsELF3-2, an ortholog of the *Arabidopsis* ELF3, and promotes their degradation via the 26S proteasome pathway to positively regulate basal defense against *M*. *oryzae* (You et al., 2022, 2023). OsPEX5 was further found to stabilize the aldehyde dehydrogenase OsALDH2B1 to enhance its repression of the defense-related gene OsAOS2. The Bowman-Birk trypsin inhibitor (BBTI) APIP4 functions as a positive regulator of rice blast resistance (Zhang et al., 2020a). The interaction between AvrPiz-t and APIP4 suppress its trypsin inhibitor activity, while the binding of APIP4 with Piz-t potentially promotes the activity of APIP4, resulting in enhanced rice immunity. Like APIP4, APIP5 is the target of both AvrPiz-t and Piz-t. It plays a critical role in preventing effector-triggered necrosis (ETN) during the necrotrophic stage of *M*. *oryzae* infection (Wang et al., 2016; Zhang et al., 2022). APIP5 directly targets the cell wall-associated kinase gene *OsWAK5* and the cytochrome P450 gene *CYP72A1* as a transcription factor to inhibit their expression, resulting in less lignin, ROS and defense compounds accumulation. Besides, APIP5 regulates mRNA turnover of the cell death- and defense-related genes *OsLSD1* and *OsRac1* as an RNA-binding protein. AvrPiz-t attenuates the transcriptional activity and protein accumulation of APIP5, leading to ETN at the necrotrophic stage. Piz-t can stabilize APIP5 and reduce the AvrPiz-t-mediated APIP5 turnover to prevent ETN. In turn, APIP5 is essential for the accumulation of Piz-t for the activation of ETI. A recent work showed that APIP5 directly suppresses the transcription of APIP4 and its homolog OsBBTI5, thereby attenuating their trypsin inhibitor activity to weaken the disease resistance (Zhang et al., 2024a). APIP4 and OsBBTI5 were further proved to associate and stabilize the defense-related protein OsPR1aL, which positively regulates rice blast resistance. APIP7 (OsAKT1) forms a complex with OsCBL1 and OsCIPK23, modulating K^+^ signal transduction for plant growth and development, as well as immunity (Shi et al., 2018). AvrPiz-t suppresses the activity of APIP7 and/or interferes with the APIP7-OsCIPK23 complex to subvert inward K^+^ currents in favor of *M. oryzae* pathogenesis. APIP12, targeted by both AvrPiz-t and APIP6, is involved in the basal resistance but not the Piz-t mediated resistance (Tang et al., 2017). AvrPiz-t was also found to interact with OsRac1 to suppress ROS generation (Bai et al., 2019).

## AVR effector vs. paired NLRs

The genetically and molecularly co-acting NLR pairs are prevalent in rice and other plant genomes (Duxbury et al., 2021; Xi et al., 2022). The Pia pair RGA4/RGA5 recognize two sequence-unrelated AVR effectors, AVR-Pia and AVR1-CO39 (Cesari et al., 2013). Both these two AVR effectors bind to the HMA ID integrated into the sNLR, RGA5. RGA4, as the hNLR, is autoactive and its function is repressed by RGA5 in the absence of pathogen (Cesari et al., 2014). This repression is relieved upon direct interaction of AVR-Pia or AVR1-CO39 with the HMA domain in RGA5, leading to activation of ETI (Cesari et al., 2013; Ortiz et al., 2017). Pias pair Pias-1/Pias-2, which is allelic Pia pair, detects the AVR effector AVR-Pias. Interestingly, the sNLR Pias-2 carries a different ID, DUF761, and no direct binding between AVR-Pias and DUF761 of Pias-2 was observed (Shimizu et al., 2022). For the Pik pair Pik-1/Pik-2 and its cognate AVR effector Avr-Pik, both of them exist in an allelic series in rice and *M*. *oryzae*, respectively (Yoshida et al., 2009; Kanzaki et al., 2012; Wu et al., 2014; De la Concepcion et al., 2018). At least 7 Pik alleles (Pi1, Pik, Pikm, Pikp, Piks, Pikh and Pike) and 6 AVR-Pik variants (A-F) have been reported. The Pik alleles showing different recognition specificities to AVR-Pik variants. Pik-1 recognition of AVR-Pik is mediated by direct binding of the AVR effector to a HMA domain, integrated into between the CC and NB‐ARC domains. In contrast to RGA4, the hNLR Pik-2 does not show autoimmunity in an ectopic expression system, and both NLRs are required to trigger an immune response upon perceiving the matching AVR effector (Maqbool et al., 2015). Besides, AVR-Pik variants interact with a subset of small HMA‐containing (sHMA) protein, which belong to heavy metal-associated plant proteins (HPPs) and heavy metal-associated isoprenylated plant proteins (HIPPs) (Maidment et al., 2021; Oikawa et al., 2024). AVR-PikD binds and stabilizes OsHIPP19 and OsHIPP20 in plant cells. The binding affects the subcellular distribution of the OsHIPP19 and OsHIPP20. Knockout of OsHIPP20 conferred enhanced resistance to infection by the blast pathogen, suggesting OsHIPP20 is a susceptibility gene. Therefore, it is hypothesized that AVR-Pik-mediated stabilization of sHMA proteins suppresses rice defenses. Additionally, AVR-Mgk1, an effector sharing no sequence similarity to known AVR-Pik family, is found on a mini-chromosome and detected by Piks as well as other multiple Pik alleles (Sugihara et al., 2023). Recent studies reported that Avr-PikD interacts with the zinc finger−type transcription factor WG7 and the LSD1-like transcription factor AKIP30. WG7 negatively regulates immunity through SA signaling pathway (Yang et al., 2024). Avr-PikD suppresses rice immunity by targeting WG7 in nucleus and promoting its transcriptional activity. By contrast, AKIP30 is also a positive regulator of rice immunity. Avr-PikD interferes with the expression, subcellular localization and transcriptional activity of AKIP30, thereby facilitating ETS (Guo et al., 2024). AVR-Pii interacts with two members of rice Exo70 family, OsExo70F2 and OsExo70F3, suggesting that the pathogen may target exocyst-mediated trafficking as a virulence-associated mechanism (Fujisaki et al., 2015). Exo70, a component of the exocyst complex, plays crucial roles in tethering and fusion of the vesicles and plasma membrane at the site of polarized exocytosis (Munson and Novick, 2006). It was revealed that OsExo70F3 is specifically involved in Pii-dependent resistance (Fujisaki et al., 2015). The association of AVR-Pii with OsExo70F3 is monitored by Pii through an unconventional RIN4/NOI domain integrated in the sNLR Pii-2 (Fujisaki et al., 2017). AVR-Pii also targets OsNADP-ME2, a rice nicotinamide adenine dinucleotide phosphate-malic enzyme, and inhibit its activity to limit ROS accumulation and suppress basal resistance (Singh et al., 2016).

## AVR effector vs. uncharacterized R protein

Even though PWL2 is capable of being recognized by the NLR protein Mla3 in barely which confers resistance to *Blumeria graminis* and *M*. *oryzae*, its corresponding R protein in rice has not yet been identified (Brabham et al., 2024). More recently, it was reported PWL2 specifically binds to HIPP43 in rice and its orthologs from other grass species (Were et al., 2024; Zdrzałek et al., 2024). HIPP43 is a susceptibility factor for infection, since overexpression of HIPP43 suppresses PAMP-induced ROS in transgenic plants. PWL2 targets HIPP43 to stabilize and alter plasmodesmata localization of HIPP43, thus enhancing susceptibility (Were et al., 2024). ACE1, coding for a PKS-NRPS hybrid, is the only non-secreted AVR effector in *M*. *oryzae* to date (Böhnert et al., 2004). It is located in a secondary metabolite gene cluster exclusively expressed during fungal appressorium-mediated penetration (Collemare et al., 2008). The AVR signal detected by the R protein Pi33(t) is not the ACE1, but the secondary metabolite synthesized by it. However, the expression of ACE1 is under strict temporal and cell type-specific regulation and its produced secondary metabolite is extremely difficult to isolate. Ectopic expression of ACE1 indicated that the metabolite is likely to be a tyrosine-derived cytochalasan compound (Song et al., 2015). But the exact AVR molecule remains to be determined.

## Structural overview of *M. oryzae* AVR effectors and their interactions with rice R/target proteins

In natural pathosystems, AVR effectors are under strong selection pressure to adapt to specific or new hosts and evade immunity, which has driven their rapid expansion and diversification (Fouché et al., 2018). The majority of fungal AVR effectors share low sequence similarity with each other or with other proteins of known function (Ellis et al., 2009). Therefore, the prediction on their function is challenging. A protein’s three-dimensional structure can provide key insights into function and evolution. As such, structural determination has become an avenue pursued to understand roles of the effectors in the infection process. Till now, the 3-dimensional structures of seven AVR effectors including AvrPiz-t, AVR-Pia, AVR1-CO39, AVR-Pik variants, AvrPib, AVR-Pii and PWL2 have been experimentally solved (Zhang et al., 2013; De Guillen et al., 2015; Maqbool et al., 2015; Ose et al., 2015; Zhang et al., 2018; De la Concepcion et al., 2022). All these AVR effectors except AVR-Pii belong to the MAX (*Magnaporthe* AVRs and ToxB-like) effector family, which accounts for 5-10% of the effector repertoire in *M*. *oryzae* (De Guillen et al., 2015; Kotsaridis et al., 2023). The crystal structure of AVR-Pii/OsExo70F2 complex revealed a fold for AVR-Pii based on a zinc-finger (ZiF) motif sustained by four residues coordinating a Zn^2+^ atom and the structure has not been previously reported for other phytopathogen effectors. AVR-Pii binds to Exo70 via a conserved hydrophobic pocket (De la Concepcion et al., 2022). MAX effectors share a common fold with six-stranded β-sheet sandwich and the fold is stabilized by at least one disulfide bond between conserved cysteins connecting β1 and β5 (De Guillen et al., 2015). Even containing the similar structure, distinct shapes and surface properties due to the varying orientation and length of β-strands and loops constitute the basis of diversity in their functions. For example, AvrPib and AvrPiz-t contain the shorter β-strand β6 at the C-terminus, while the shorter one of AVR1-CO39, AVR-Pia and AVR-Pik is β5 (Zhang et al., 2018). AVR1-CO39, AvrPiz-t and AvrPib have dominant charge patch(es) on the surfaces, but AVR-Pia and AVR-Pik have only hydrophobic patch with multiple charged residues distributed separately on the surfaces.

## Bioengineering of rice NLRs guided by structural knowledge of NLR-AVR interactions

Management of rice blast disease is cumbersome, even though rice *R* genes have been extensively used in breeding (Wang and Valent, 2017; Younas et al., 2023). The recognition spectra of R proteins tend to be specific and *M*. *oryzae* may delete AVR effectors from their genome or evolve novel AVR variants that evade detection by the R proteins to re-establish infection (Maekawa et al., 2011). With increasing mechanistic and structural insights into the NLR-ID-AVR interactions, bioengineering of NLR’s ID has emerged as a promising approach to expand its recognition specificities. Recent studies have reported that HMA domain engineering is an effective way to generate new resistance specificities. A binding interface was grafted from Pikm-1-HMA onto Pikp-1-HMA by mutating two residues in Pikp-1 and the engineered variant gained an expanded recognition profile to AVR-Pik variants previously unrecognized by Pikp in *N*. *benthamiana* (De la Concepcion et al., 2019). Introduction of the HMA or three specific residues in the interface of OsHIPP19 into Pikp-1-HMA creates Pikp-1 variants that recognize all known AVR-Pik alleles including AVR-PikC and AVR-PikF, which are not detected by naturally occurring Pik-1, not only in *N*. *benthamiana* but also in rice (Maidment et al., 2021). Integration of the HMA of OsHIPP43 into the Pikm-1 switches recognition from AVR-Pik to PWL2, as well as PWL1 and PWL3 in *N*. *benthamiana* (Zdrzałek et al., 2024). By combining the AVR-PikD binding residues of Pikp-1-HMA into RGA5-HMA, a variant gained an extended resistance specificity in *N*. *benthamiana* but not in transgenic rice (Cesari et al., 2022). The modified sites may affect NLR activation or additional interactions with RGA5 outside the ID might be important for recognition. In another two studies, RGA5-HMA was engineered by comparing the structures of AVR1-CO39 and the noncorresponding AVR-Pib and AVR-PikD for predicting their potential interface. The engineered RGA5 confers specific resistance to *M*. *oryzae* strains expressing AvrPib or AVR-PikD in transgenic rice (Liu et al., 2021b; Zhang et al., 2024b). More recently, a groundbreaking approach for molecular engineering of Pikm-1 by replacing HMA ID with camelid-derived nanobodies of fluorescent proteins (FP) was reported (Kourelis et al., 2023). The synthetic Pikm-1s with nanobodies trigger HR in the presence of Pikm-2 and the corresponding fluorescent proteins in *N*. *benthamiana* and confer resistance against plant viruses expressing FPs. These studies collectively demonstrated the potential for engineering IDs to alter the recognition profiles of the NLR proteins.

## Conclusion and future perspectives

Over the past three decades, despite our understanding the roles of AVR effectors of *M*. *oryzae* in establishing interactions with rice and other hosts is increasing, many issues and challenges (listed below) remain to be resolved:

(1) The AVR effectors corresponding to the majority of known R proteins, particularly those with broad-spectrum resistance, such as Pigm, Pi2, etc., have not yet been isolated. These AVR effectors may be highly conserved and prevalent across *M*. *oryzae* population in the field. The loss of them likely imposes fitness penalties on the pathogen (Leach et al., 2001; Bart et al., 2012).
(2) Since wheat is currently threatened by the expanding blast pandemic, research efforts are urgent to isolate more AVR effector and R protein pairs. It will enable the study of their molecular interactions and the potential for engineering resistance against the *Triticum* pathotype of *M*. *oryzae*.
(3) What is the final product synthetized by ACE1 and how does Pi33 detect the AVR signal?
(4) The structural mechanism underlying the transformation of NLRs from their static to activated states upon recognition of AVR effectors needs further investigation.
(5) What are the detailed molecular events downstream once R protein is activated by AVR effectors?
(6) Little is known about the detailed mechanism by which the cytoplasmic AVR effectors are internalized and transported into plant cells. Once entering into the cytoplasm, how these AVR effectors move into the cellular organelles for virulence and avirulence functions remains to be addressed.

Future research in these fields will undoubtedly reveal novel strategies of *M*. *oryzae* AVR effectors participating in rice resistance/susceptibility that can be exploited to control blast disease with high efficiency and durability.
